# Screening and Bioinformatics Analysis of Competitive Endogenous RNA Regulatory Network ––Related to Circular RNA in Breast Cancer

**DOI:** 10.1155/2021/5575286

**Published:** 2021-09-10

**Authors:** Tao Wang, Yi Zhang, Yan He, Yang Liu, Peng Qi

**Affiliations:** ^1^Department of Thyroid and Breast Surgery, Hubei No. 3 People's Hospital of Jianghan University, Wuhan 430000, China; ^2^Department of Endocrinology, Hubei No. 3 People's Hospital of Jianghan University, Wuhan 430000, China

## Abstract

**Purpose:**

Circular RNA as a competitive endogenous RNA (ceRNA) plays a significant role in the pathogenesis and progression of breast cancer. In this study, a circular RNA-related ceRNA regulatory network was constructed, which provides new biomarkers and therapeutic targets for the treatment of breast cancer. *Materials and methods*. The expression profile datasets (GSE101123, GSE143564, GSE50428) of circRNAs, miRNAs, and mRNAs were downloaded from the GEO database, and then differentially expressed RNAs (DEcircRNAs, DEmiRNAs, DEmRNAs) were obtained through the CSCD, TargetScan, miRDB, and miRTarBase databases. CircRNA-miRNA pairs and miRNA-mRNA pairs were constructed. Finally, a ceRNA regulatory network was established. Downstream analysis of the ceRNA network included GO, KEGG analysis, survival analysis, sub-network construction, the BCIP, and qRT-PCR verification.

**Results:**

In total, 144 differentially expressed (DE) DEcircRNA, 221 DEmiRNA, and 1211 DEmRNA were obtained, and 96 circRNA-miRNA pairs and 139 miRNA-mRNA pairs were constructed by prediction. The ceRNA regulatory network (circRNA-miRNA-mRNA) was constructed, which included 42 circRNA, 36miRNA, and 78 mRNA. GO function annotation showed genes were mainly enriched in receptor activity activated by transforming growth factor beta (TGF-beta) and in the regulation of epithelial cell apoptosis. KEGG analysis showed genes were mainly enriched in the TGF-beta signaling, PI3K-Akt signaling, and Wnt signaling pathways. Four genes associated with survival and prognosis of breast cancer were obtained by survival analysis, the prognostic sub-network included 4 circRNA, 4 miRNA, and 4 mRNA. BCIP analysis and qRT-PCR verification confirmed that relative mRNA expression levels were consistent with those in the GEO database.

**Conclusion:**

A circRNA-related ceRNA regulatory network was constructed for breast cancer in this study and key genes affecting pathogenesis and progression were identified. These findings may help better understand and further explore the molecular mechanisms that affect the progression and pathogenesis of breast cancer.

## 1. Introduction

Breast cancer is the most common malignancy and poses a serious threat to the health of women worldwide. In 2020, new cases of breast cancer worldwide accounted for 30% of all malignant tumors, and it is also the second leading cause of cancer-related deaths among women [1]. Despite the continuous development of medical technology and the decline in mortality rates, the incidence of breast cancer is still increasing worldwide [2]. Breast cancer is a systemic disease, and its treatment methods include surgery, chemotherapy, targeted therapy, radiotherapy, immunotherapy, and neoadjuvant therapy. Although there are various treatment methods, the mortality rate of breast cancer remains high due to the high recurrence rate, distant metastasis, and drug resistance. Studies have shown that gene mutations and gene disorders are the main reasons leading to the occurrence and progression of many cancers [3]. Therefore, there is an urgent need for new biomarkers and therapeutic targets for breast cancer.

In 1976, researchers identified circular RNA genome in a virus [4]. Since then, studies have shown that circular RNA plays a crucial role in biological processes [5, 6]. The rapid development of high-throughput microarray technology has allowed researchers to analyze the expression profiles of circular RNAs, which in turn has facilitated research into tumor-related genes and further exploration of the molecular mechanisms involved [7–9]. Circular RNA has a closed loop structure without a 5′-end cap and 3′-poly(A) tail [10]. When circRNA was first discovered, it had been mistakenly regarded as a byproduct of splicing, but it is now considered a key molecule in many biological processes [11]. Studies have shown that circRNA is involved in the onset and progression of many diseases, including a variety of cancers [12]. For instance, circHIPK3 as a molecular sponge of miR-558 inhibits the invasion and metastasis of bladder cancer [13]. Studies have also suggested that circFBLIM1 can promote the development of hepatocellular carcinoma, and it may be used as a therapeutic target for hepatocellular carcinoma [14]. In breast cancer, certain circRNAs have also been identified as oncogenes or tumor suppressor genes [15, 16].

The databases and bioinformatics update provide resources and methods for studying the regulation of non-coding RNA [17–19]. Pandolfi et al. devised the competitive endogenous RNA hypothesis (ceRNA), which proposed that long non-coding RNA, mRNA, and other transcripts can competitively bind miRNA response elements (MREs) to mutually regulate the expression of their respective genes, revealing a novel mechanism of RNA interaction [20]. CircRNA, which belongs to the non-coding RNA family can also competitively adsorb miRNAs, thereby regulating gene expression at the post-transcriptional level [5, 21]. Deng et al. found that circRHOBTB3 can competitively bind miR-654-3p to inhibit the progression of gastric cancer [22]. CircGFRA1 as a ceRNA can be used to regulate GFRA1 by competitively binding miR-34a, and thereby, plays a regulatory function in triple-negative breast cancer (TNBC) [23]. Therefore, circRNA may become a potential therapeutic target and biomarker for malignant tumors. However, the biological function of most circRNA and their molecular mechanisms in breast cancer are still unclear.

**Figure 1:**
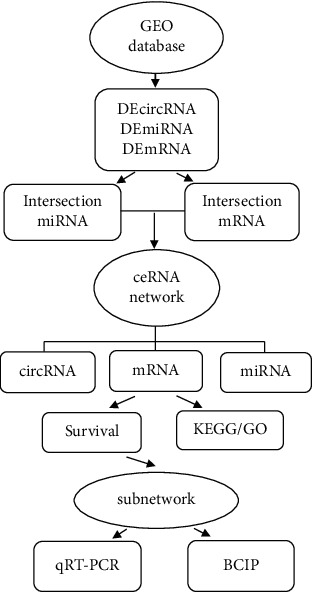
Flow chart for the construction of circRNA-related ceRNA regulatory network.

In our study, three datasets, downloaded from the Gene Expression Omnibus (GEO) database, were grouped and analyzed to obtain the differentially expressed (DE) circRNA, miRNA, and mRNA (DEcircRNA, DEmiRNA, and DEmRNA, respectively). The circRNA-related ceRNA regulatory network was constructed through predictions derived from the cancer-specific circRNA database (CSCD) database, miRDB, and TargetScan database. GO analysis, KEGG analysis, and survival analysis was performed on mRNA in the ceRNA network, and a prognostic sub-network was constructed. Finally, mRNA expression of prognostic-related genes was verified using the Breast Cancer Integrated Platform (BCIP) and quantitative polymerase chain reaction (qRT-PCR) assays. CircRNA, miRNA, and mRNA correlating with the prognosis of breast cancer were discovered, which will help to further deepen our understanding of the molecular mechanism and biological process of breast cancer. The flowchart of this research is shown in Figure 1.

## 2. Materials and Methods

### 2.1. Data Collection

The circRNA, miRNA, and mRNA datasets were downloaded from the GEO database for subsequent differential expression analysis and construction of the ceRNA network. GEO (https://www.ncbi.nlm.nih.gov/gds/) is a public database of genomes. Three datasets (GSE101123, GSE143564, GSE50428, Platforms information: Agilent-0699900 Arraystar Human CircRNA microarray V1, [miRNA-4] Affymetrix Multispecies miRNA-4 Array [ProbeSet ID version], Agilent-021412 nONCOchip_1.0 021253) related to breast cancer were downloaded from the GEO database in this study. The GSE101123 contains eight breast cancer tissues and three normal breast tissues. The GSE143564 contains three breast cancer tissues and paired normal adjacent tissues. And the GSE50428 contains 26 breast cancer tissues and five normal breast tissues.

### 2.2. Identification of DEcircRNA, DEmiRNA, DEmRNA Were Obtained

The genes used to construct the ceRNA network must be differentially expressed. Perl software version 5.30.0.1 was used to convert the probe matrix into an RNA matrix. DEcircRNA, DEmiRNA, and DEmRNA were obtained using the limma package in R version 4.0.3. Screening criteria: ∣log_2_ fold change∣ >1 and adjusted P-value <0.05 were considered statistically significant. The pheatmap package in R is used for the visual analysis of differentially expressed RNA.

### 2.3. Construction of the ceRNA Network

We constructed a circRNA-miRNA-mRNA regulatory network based on the ceRNA theory. CircRNA information was derived from the circBase database (http://www.circbase.org/cgi-bin/getseq.cgi). Then, the CSCD (http://gb.whu.edu.cn/CSCD/) was used to predict the target miRNAs of DEcircRNA. The Venn package was used to intersect these target miRNAs with the DEmiRNA in the GSE143564 dataset, then intersection miRNAs were obtained, and finally, circRNA-miRNA pairs were established. Through TargetScan (http://www.targetscan.org/vert_71/), miRDB (http://mirdb.org/), and miRTarBase (http://mirtarbase.cuhk.edu.cn/php/index.php) databases, the target mRNAs of intersection miRNAs are predicted, and only genes identified in the above three databases at the same time were selected as potential target mRNAs. Likewise, these target mRNAs are intersected with DEmRNAs in the GSE50428 dataset to obtain miRNA-mRNA pairs. Finally, the data of the regulatory network (circRNA-miRNA-mRNA) was obtained through Perl software, and then a visual ceRNA regulatory network was established using Cytoscape 3.8.0 software.

### 2.4. GO Function Annotation and KEGG Pathway Analysis

To further explore the pathway and mechanism of DEmRNA in the ceRNA network affecting breast cancer, we conducted function annotation and KEGG pathway analysis. Gene Ontology (GO) function annotation (http://geneontology.org/) is a widely used bioinformatics analysis method, including molecular function, cellular components, and biological processes. The Kyoto Encyclopedia of Genes and Genomes(KEGG) database (https://www.kegg.jp/)is a widely used public resource that can be used to understand molecular-level information, biological systems, and advanced functions [24]. The http://org.Hs.eg.db package in R was used to convert gene symbols to gene IDs. The clusterProfiler, enrichplot, colorspace, stringi, DOSE, and ggplot2 packages in R were used to complete GO analysis and KEGG pathway analysis.

### 2.5. Survival Analysis

We conducted a survival analysis to explore whether DEmRNA in the ceRNA network affects the overall survival of breast cancer patients. The Cancer Genome Atlas (TCGA) (https://www.cancer.gov/about-nci/organization/ccg/research/structural-genomics/tcga) is currently the world's largest public database of cancer genetic information. It aims to help researchers understand cancer more deeply, thereby improving cancer prevention, diagnosis, and treatment capabilities by means of high-throughput genome analysis technology. The TCGA database contains gene expression data, miRNA expression data, frequency, and standardized clinical data with large sample sizes. Perl software was used to extract the information in the TCGA database, and then survival and survminer packages are used to analyze the relationship between target genes and the survival rate of patients to obtain mRNAs related to the survival and prognosis of breast cancer. Then, using Cytoscape software, a prognostic sub-network was constructed for the obtained prognostic-related genes, and then the miRNA and circRNA exerting a regulatory relationship with them were confirmed.

### 2.6. Relative mRNA Expression Level by qRT-PCR

QRT-PCR was used to detect the relative expression levels of mRNA (TPD52, BTG2, CCND2, LIFR) in human breast gland cell line MCF10A and breast cancer cell line MCF-7. Primer sequence information for the PCR experiment is shown in Table 1. The above cell lines were purchased from the Shanghai Cell Bank of the Chinese Academy of Sciences. The real-time fluorescent quantitative PCR instrument (Bio-Rad iQ5) was used. Beta-actin was used as an internal reference, and the reaction procedure adopted a two-step method. The reaction conditions: pre-denaturation at 95°C for 10 min; denaturation at 95°C for 15 seconds, annealing/extension at 60°C for 60 seconds, 42 cycles. The Ct value of each sample is derived through analysis, and the relative expression level of mRNA was determined by calculating the 2^-△△Ct^ value.

### 2.7. The Expression Value of mRNAs in BCIP

Through the BCIP database (using breast cancer tissues and adjacent normal breast tissues), we further verified the expression levels of candidate genes. The BCIP (http://www.omicsnet.org/bcancer/database) is a website that analyzes and visualizes the genes of breast cancer patients. The platform's data comes from public databases, including the TCGA database, GEO database, and the Molecular Taxonomy of Breast Cancer International Consortium (METABRIC). The BCIP database can be used for multi-omics comprehensive analysis, by which we have obtained the expression value of 4 mRNAs in adjacent normal tissues and breast cancer tissues.

## 3. Results

### 3.1. DEcircRNA, DEmiRNA, and DEmRNA Were Extracted

**Figure 2:**
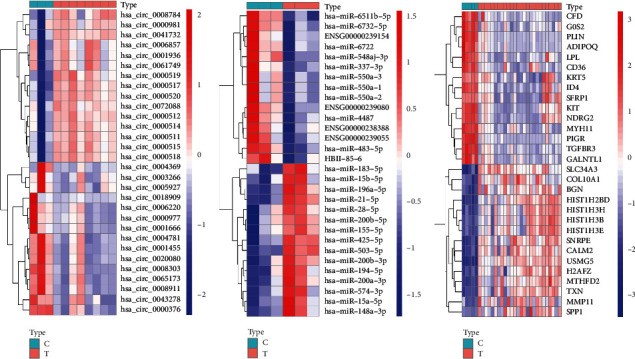
Heatmap of differentially expressed circRNA, miRNA, and mRNA. Red represents upregulated expression, and green means downregulated expression. (a) 144 differentially expressed circRNAs in the GSE101123 dataset. (b) 221 differentially expressed miRNAs in the GSE143564 dataset. (c) 1211 differentially expressed mRNAs in the GSE50428 dataset (|log_2_ fold change|>1，adjusted P-value<0.05).

Human-derived RNA datasets (GSE101123, GSE143564, and GSE50428) containing breast cancer tissues and normal breast tissues were selected. The differential expression analysis of datasets was carried out through the limma package, ∣log_2_ fold change∣ >1, and the adjusted P-value was <0.05. In total, 144 DEcircRNAs were obtained, of which 64 were downregulated and 80 were upregulated. Of the 221 DEmiRNAs, 97 were downregulated and 124 were upregulated. There were 1211 DEmRNAs, 713 were downregulated and 498 were upregulated. We selected the top 15 downregulated and upregulated DERNAs for heat map analysis, as shown in Figure 2. Basic information of the three datasets is shown in Table 2.

### 3.2. Identification of Target miRNA and mRNA of CircRNA

**Figure 3:**
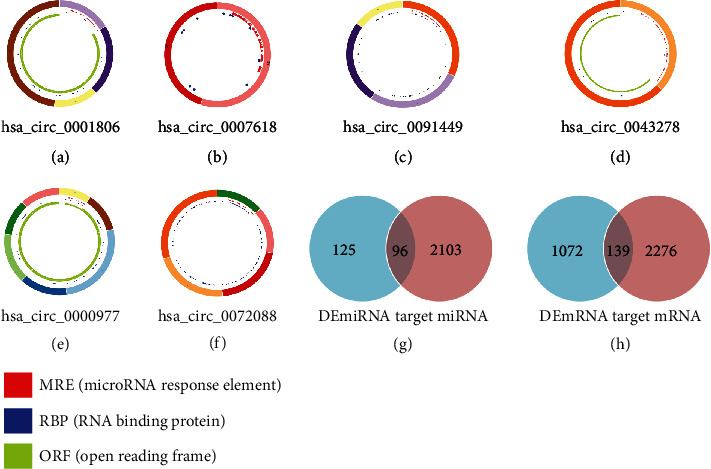
Structural patterns of circRNAs and Venn diagram of RNAs involved in ceRNA network. (a) hsa_circ_0001806. (b) hsa_circ_0007618. (c) hsa_circ_0091449. (d) hsa_circ_0043278. (e) hsa_circ_0000977. (f) hsa_circ_0072088. (g) We confirmed 96 intersection miRNAs by intersecting target miRNA from CSCD and differentially expressed miRNAs (DEmiRNA) obtained from GEO. (h) We confirmed 139 intersection mRNAs by intersecting target mRNA from the miRNA target gene prediction website and differentially expressed mRNAs (DEmRNA) obtained from GEO.

CircRNA can combine with miRNA response elements (MREs) to achieve a regulatory role. Through the CSCD database, we predicted a total of 2199 target miRNAs that bind to DEcircRNA. However, in the CSCD database, we did not find any information about any of these 12 circRNAs including hsa_circ_0001625 et al. The structure pattern diagrams of 6 circular RNAs, are shown in Figures 3(A)-3(F). Subsequently, using the Venn diagram method, the predicted target miRNA and DEmiRNA were intersected, and 96 intersection miRNAs were obtained, as shown in Figure 3g. Next, through the TargetScan, miRTarBase, and miRDB databases, only genes identified in the above three databases at the same time were selected as potential target mRNAs, then, the above-mentioned intersection miRNAs were predicted to obtained target 2415 mRNAs. By intersecting the target mRNA and DEmRNA, 139 intersection mRNAs were obtained, as shown in Figure 3h.

### 3.3. Construction of ceRNA Regulatory Network

**Figure 4:**
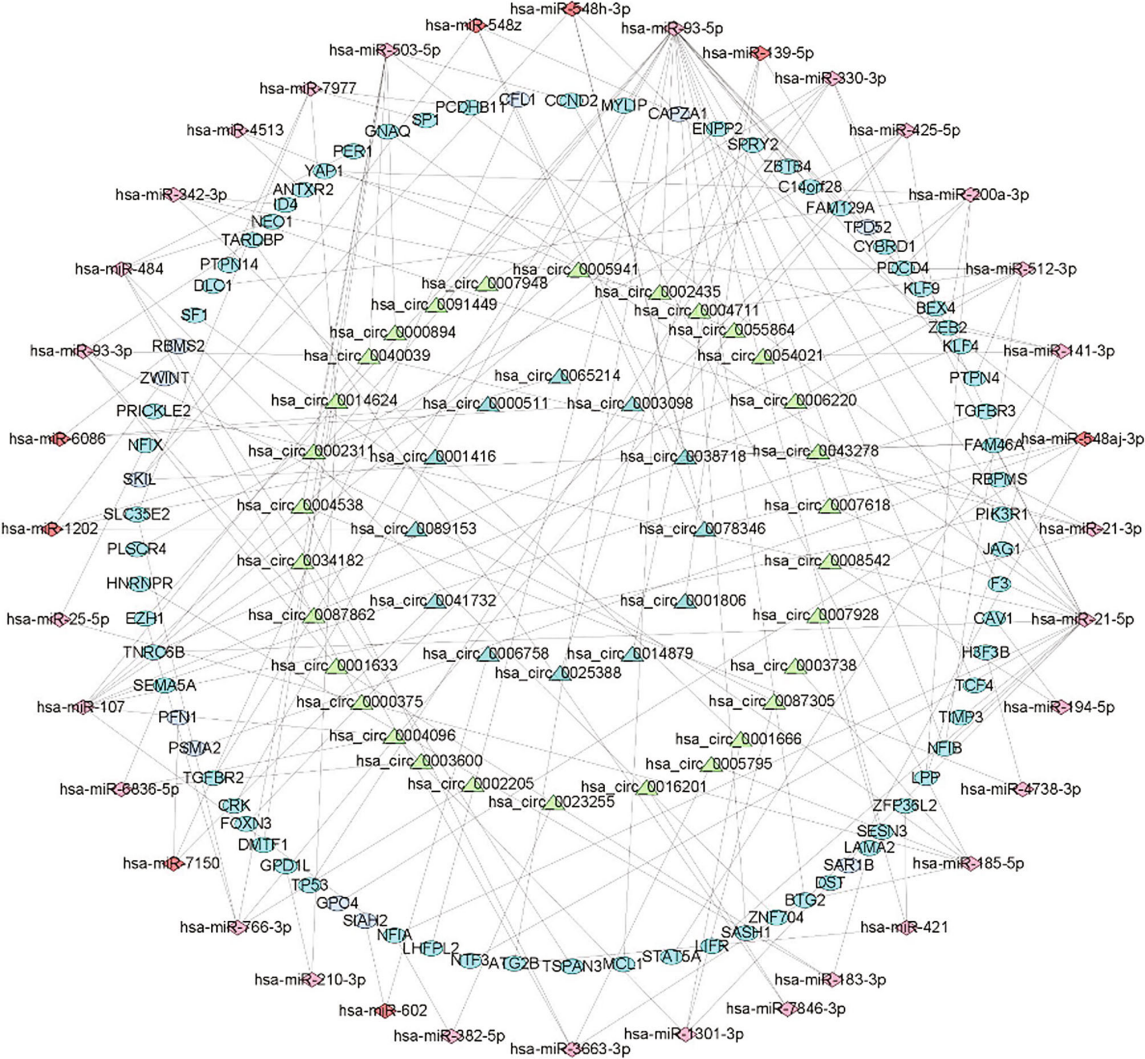
The visualized ceRNA regulatory network was constructed by Cytoscape software. The triangle represents 42 circRNAs, the diamond represents 36 miRNAs and the ellipse represents 78 mRNAs.

**Figure 5:**
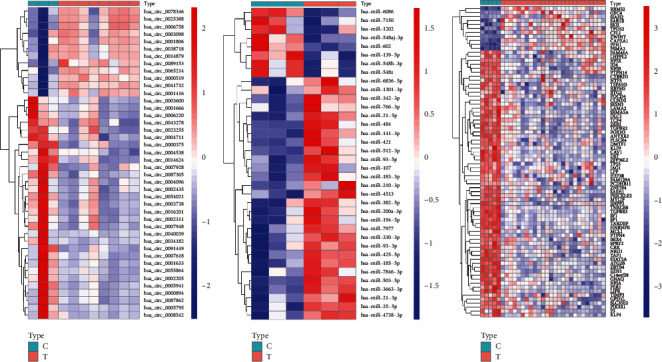
Heatmap of circRNA, miRNA, and mRNA in the ceRNA regulatory network. (a) 42 circRNAs. (b) 36 miRNAs. (c) 78 mRNAs.

Only genes that meet the following conditions will be selected into the ceRNA network:

(1) All genes must be differentially expressed; (2) circRNAs and mRNAs have a binding relationship with miRNAs at the same time; (3) RNAs (circRNA, mRNA) and miRNAs that meet the above binding relationship must be negatively regulated. Then, through Cytoscape software, a ceRNA regulatory network (circRNA-miRNA-mRNA) was constructed, including 42 circRNA, 36 miRNA, and 78 mRNA (Figure 4). Meanwhile, the pheatmap package in R was used to visualize the circRNA, miRNA, and mRNA in the ceRNA regulatory network (Figures 5(a)-5(c))

### 3.4. GO Function Annotation and KEGG Pathway Analysis

**Figure 6:**
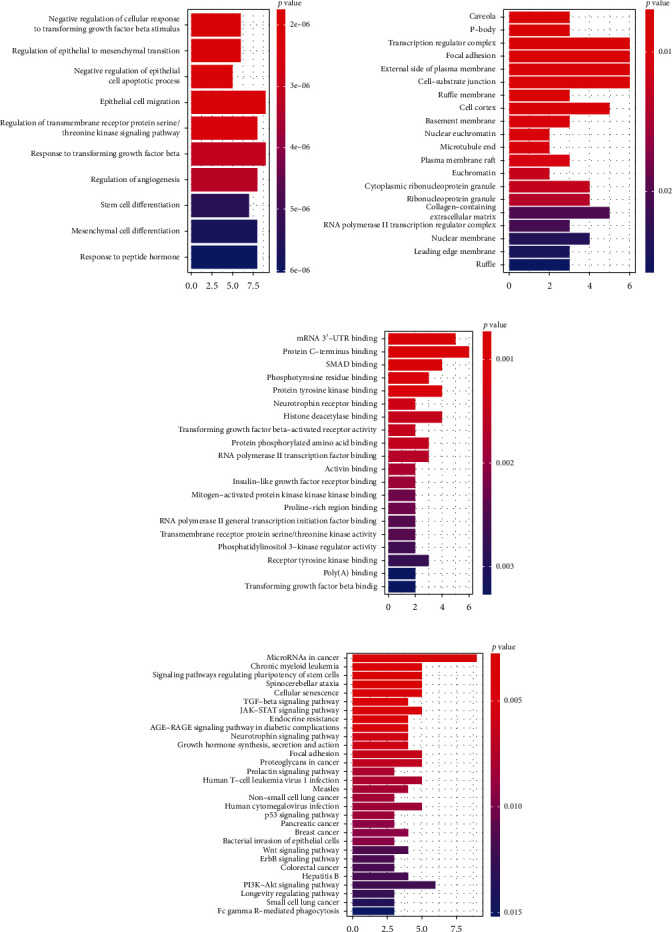
The GO and KEGG enrichment analysis of mRNA in the ceRNA network. (a) The top 10 GO terms of biological process. (b) The top 20 GO terms of cellular component. (c) The top 20 GO terms of molecular function. (d) The top 30 KEGG pathways.

In the ceRNA regulatory network, GO analysis of 78 mRNAs showed that they are mainly enriched in: negative regulation of cellular response to transforming growth factor beta stimulus, regulation of epithelial to mesenchymal transition (EMT), regulation of epithelial cell apoptotic process, positive regulation of phosphatidylinositol 3-kinase (PI3K) signaling (Biological Process BPs), transcription regulator complex (Cellular Component CCs), transforming growth factor beta-activated receptor activity, kinase regulator activity, protein tyrosine kinase binding, and transcription corepressor activity (Molecular Function MFs). The histogram is shown in Figures 6(a)-6(c). KEGG pathway analysis results show mainly enrichment in the PI3K-Akt signaling pathway, TGF-beta signaling pathway, Wnt signaling pathway, ErbB signaling pathway, p53 signaling pathway, and in microRNAs in cancer (Figure 6(d)). Most of the pathways identified above are involved in the incidence and progression of breast cancer.

### 3.5. Survival Analysis and Prognostic Sub-Network Construction

**Figure 7:**
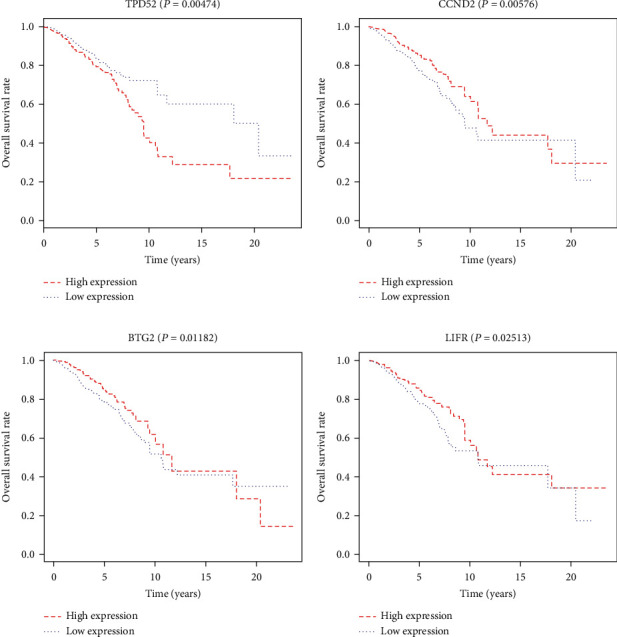
Survival curve analysis of mRNA in ceRNA network. (a-d) Survival curves showing TPD52, CCND2, BTG2, and LIFR related to an overall survival rate of breast cancer. The TPD52 gene high expression group has a worse prognosis compared with the low expression group (P =0.00474). The CCND2, BTG2, and LIFR gene high expression groups have a better prognosis compared with the low expression groups, respectively (P =0.00576, P =0.01182, P =0.02513).

**Figure 8:**
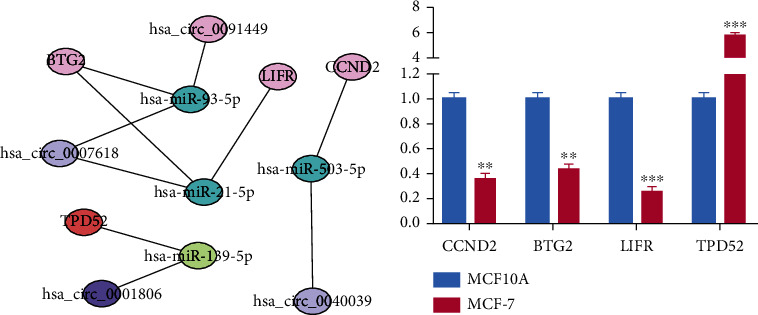
Construction of the circRNA-miRNA-mRNA sub-network and the relative expression level of mRNAs involved in the prognosis of breast cancer is verified. (a) The prognostic sub-network of ceRNA includes 4 circRNAs, 4 miRNAs, and 4 mRNAs. (b) The relative expression levels of 4 mRNAs (TPD52, CCND2, BTG2, and LIFR) in human breast cell line MCF10A and breast cancer cell line MCF-7, respectively.

The survival package in R was used for survival analysis of all 78 mRNAs in the ceRNA regulatory network, and 4 mRNAs (TPD52, BTG2, CCND2, LIFR) were identified as being significantly correlated with breast cancer survival prognosis (Figures 7(a)-7(d)). Meanwhile, based on the above four mRNAs, a circRNA-miRNA-mRNA prognostic sub-network was constructed (Figure 8(a)).

### 3.6. qRT-PCR Verification of mRNA Expression

We verified the relative expression levels of 4 mRNAs identified in the above analysis in cell lines via qRT-PCR assays. Compared with the MCF-10A cell line, the relative expression levels of BTG2, CCND2, and LIFR mRNA were significantly downregulated in the MCF-7 cell line, while TPD52 was significantly upregulated (P <0.01) (Figure 8(b)).

### 3.7. The Expression of Four mRNAs Was Evaluated in the BCIP

**Figure 9:**
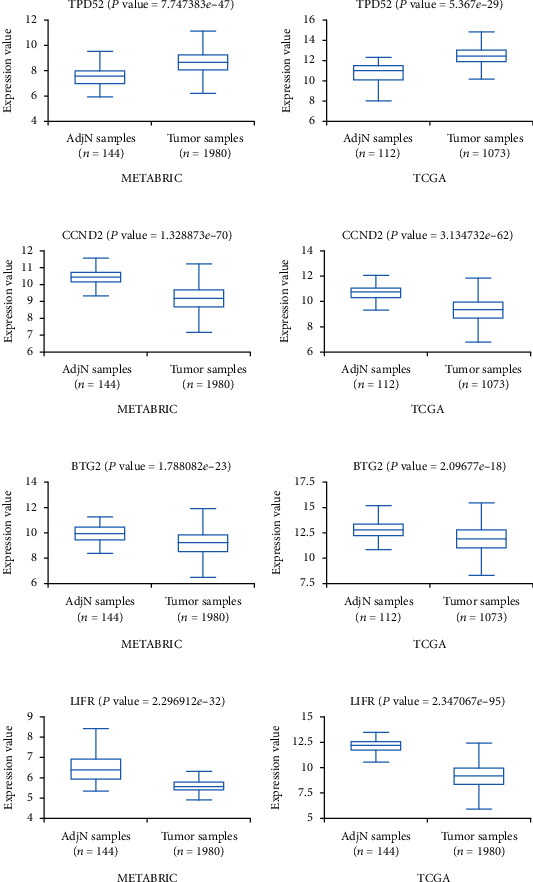
The expression values of 4 mRNAs in the BCIP database. (a-d) The expression values of TPD52, CCND2, BTG2 and LIFR in the METABRIC database and TCGA database, respectively.

Through the analysis of breast cancer data in the BCIP, the results show that the expression value of the four mRNAs in adjacent normal tissues (AdjN) and breast cancer were consistent with the GEO database (Figure 9). The median expression values of the four mRNAs are shown in Table 3.

## 4. Discussion

In recent years, researchers from different countries have made significant progress in breast cancer research, especially in the development of molecularly targeted drugs and antibody-drug conjugates, which have brought great benefits to breast cancer patients [25–28]. The current treatment methods for breast cancer include surgery, chemotherapy, endocrine therapy, molecularly targeted therapy, and radiotherapy. Although there are various treatment methods, most breast cancers achieve advanced stages, where the overall treatment effect is not satisfactory due to factors such as drug resistance, distant metastasis, and poor subtyping. The pathogenesis and progression of cancer are closely associated with gene mutations and gene disorders. Studies have shown that dysregulation of circular RNA expression plays an important role in the pathogenesis and progression of many tumors. CircRNA-related ceRNA regulatory networks play a vital role in the pathogenesis and progression of bladder cancer, colorectal cancer, and other cancers [29–31]. To date, the role and mechanism of circRNA are still unclear in breast cancer.

In this study, we used a bioinformatics analysis approach to construct a ceRNA regulatory network to identify prognostic markers for breast cancer. First, three breast cancer-related datasets, GSE101123, GSE143564, and GSE50428, were downloaded from the GEO database and were used for analysis to obtain differentially expressed RNAs (DEcircRNAs, DEmiRNAs, and DEmRNAs) in breast cancer patients. The circRNA-miRNA pairs and miRNA-mRNA pairs were obtained by predictive analysis defined by the CSCD, miRDB, miRTarget, and miRTarbase databases. Next, the ceRNA regulatory network was constructed and analyzed in function of GO and KEGG pathway analysis. The results of GO functional annotation showed that the mainly enriched genes were involved in the regulation of epithelial cell apoptotic process, regulation of EMT, negative regulation of cellular response to TGF-beta stimulus, positive regulation of PI3K signaling (BPs), transcription regulator complex (CCs), kinase regulator activity, TGF-beta-activated receptor activity, protein tyrosine kinase binding, and transcription corepressor activity (MFs). Studies have shown that the biological processes involved in EMT play a role in the progression of pancreatic cancer, malignant melanoma, and other tumors [32–37]. Further TGF-beta has an important regulatory role in the progression of multiple tumors [38–40]. The KEGG pathway analysis results indicated that the main enrichment of differentially expressed genes was in the ErbB, Wnt, TGF-beta, PI3K-Akt, and p53 signaling pathways, and in microRNAs in cancer. The involvement of these pathways is supported by previous studies. Hoxhaj et al. found the PI3K-AKT signaling pathway could directly or indirectly regulate nutrient transport and metabolic enzymes to control the metabolism of cancer cells [41]. Schmid et al. determined that the AKT inhibitor capivasertib enabled patients with TNBC to achieve longer progression-free survival and overall survival [42]. Guo B et al. found that the expression of the micropeptide protein CIP2A-BP encoded by lnc00665 was down-regulated through the TGF-beta signaling pathway, thereby inhibiting the progress of TNBC [43]. Castagnoli et al. found that WNT signaling regulated the expression of PD-L1 in the TNBC stem cell compartment [44].

We identified 4 mRNAs (TPD52, BTG2, CCND2, LIFR) involved in the prognosis of breast cancer using survival analysis. The involvement of these genes in cancer progression is supported by previous studies. Fu et al. reported that TPD52, targeted by mir-103a-3p, could affect the progression of salivary adenoid cystic carcinoma [45]. Further, Shi et al. found that the miR-185-5p/TPD52 axis was associated with radiation sensitivity of TNBC [46]. The researchers found that BTG2 is closely associated with the metastasis and progression of non-small cell lung cancer and gastric cancer [47, 48]. Yu et al. found that LncRNA TUG1 could promote cisplatin resistance in bladder cancer by regulating the expression of CCND2 [49]. The dysregulation of LIFR influences the development of prostate cancer and breast cancer [50, 51].

A prognostic sub-network was constructed, and we identified miRNA and circRNA combined with mRNA that having a regulatory relationship. Studies have shown that in breast and ovarian cancer, miR-503-5p can lead to drug resistance through targeted binding with other genes, and may also affect the proliferation and apoptosis of ovarian cancer [52–54]. Regulation of miR-93-5p expression is closely associated with the progression of breast and lung cancer [55, 56]. Wang et al. found that miR-21-5p could contribute to EMT in endometrial cancer [57].

Finally, we verified the expression of mRNA in the prognostic sub-network in breast cancer tissues and breast cancer cells, respectively, using BCIP and qRT-PCR.

Our research is innovative. Our findings were based on data obtained not only from the GEO database, but from subsequent bioinformatics analyses that also combined source data from TCGA, the METABRIC database, the BCIP, and qRT-PCR. Further, TCGA comprises a large sample size and detailed information related to prognosis. Nonetheless, our research also has certain limitations. First of all, the sample size in the GEO database is not very large. Secondly, our research methods were mainly based on bioinformatics analysis, and there is a lack of follow-up experiments verifying the relevant molecular regulatory mechanisms. The results obtained were also based on currently available tools and websites. Finally, only the relationship between the mRNA and the prognosis was evaluated. However, in the end, we constructed a prognostic sub-network of mRNA and comprising dysregulated miRNA and circRNA. Our findings indirectly demonstrate that these dysregulated genes may affect the progression of breast cancer, and provides a rationale for future studies.

## 5. Conclusion

In this study, breast cancer datasets from the GEO database were analyzed and the findings formed the basis for the construction of a circRNA-related ceRNA regulatory network for breast cancer. The relationship between the genes involved in the ceRNA network and the signaling pathways that may be involved were studied in association with the prognosis of breast cancer survival. Our findings help to better understand the ceRNA network related to circular RNA in breast cancer. Furthermore, we identified genes related to the prognosis, which will provide new clues and a rationale for future studies designing therapeutic targets and biomarkers of breast cancer.

## Figures and Tables

**Table 1:** Primer sequence information of the genes for PCR experiment.

Gene	Primer	Sequence (5'to3')
TPD52	Forward	TCCTTTAGTATACGTTCCATTCAGC
	Reverse	GGCTTGGTTCCCCCTACTT
CCND2	Forward	GTGCATTTACACCGACAACTCC
	Reverse	CAGAGCAATGAAGGTCTGAGCA
BTG2	Forward	GAGGTGTCCTACCGCATTGG
	Reverse	AGCACTTGGTTCTTGCAGGT
LIFR	Forward	GCGAGCCTATACAGATGGTGG
	Reverse	CCACTGGGATGAGAATGGCAA
Beta-actin	Forward	CCTGGCACCCAGCACAAT
	Reverse	GGGCCGGACTCGTCATAC

**Table 2:** Basic information of three gene microarray datasets of breast cancer.

Data source	Series	Platform	Sample size(N/T)
circRNA	GSE101123	GPL19978	3/8
miRNA	GSE143564	GPL21572	3/3
mRNA	GSE50428	GPL13648	5/26

**Table 3:** Median expression values of the four genes in breast cancer tissues compared with adjacent normal tissues (AdjN) from METABRIC and TCGA, respectively, by Breast Cancer Integrative Platform (BCIP).

Gene	Median (METABRIC)		Median (TCGA)	
	AdjN tissues	Tumor tissues	AdjN tissues	Tumor tissues
TPD52	7.5792	8.6433	10.972	12.4048
CCND2	10.3733	9.144	10.6866	9.2848
BTG2	9.8903	9.1643	12.714	11.8921
LIFR	6.3842	5.5721	12.0969	9.1239

## Data Availability

The three datasets (GSE101123, GSE143564, and GSE50428) in this study were downloaded from the GEO database (https://www.ncbi.nlm.nih.gov/gds/?term=).

## Abbreviations

CSCD: Cancer-specific CircRNA database
GO: Gene ontology
KEGG: Kyoto encyclopedia of genes and genomes
GEO: Gene expression omnibus
BP: Biological process
CC: Cellular component
MF: Molecular function.

## References

[1] Siegel R. L., Miller K. D., Fuchs H. E., Jemal A. (2021). Cancer statistics, 2021. *CA-Cancer J. Clin. Jan*.

[2] Konduri S., Singh M., Bobustuc G., Rovin R., Kassam A. (2020). Epidemiology of male breast cancer. *Breast*.

[3] Hatano Y., Tamada M., Matsuo M., Hara A. (2020). Molecular trajectory of BRCA1 and BRCA2 mutations. *Front. Oncol. Mar*.

[4] Meng S., Zhou H., Feng Z. (2017). CircRNA: functions and properties of a novel potential biomarker for cancer. *Mol. Cancer. May*.

[5] Hansen T. B., Jensen T. I., Clausen B. H. (2013). Natural RNA circles function as efficient microRNA sponges. *Nature*.

[6] Memczak S., Jens M., Elefsinioti A. (2013). Circular RNAs are a large class of animal RNAs with regulatory potency. *Nature*.

[7] Li S. S., Teng S. S., Xu J. Q. (2019). Microarray is an efficient tool for circRNA profiling. *Briefings in Bioinformatics*.

[8] Schena M., Heller R. A., Theriault T. P., Konrad K., Lachenmeier E., Davis R. W. (1998). Microarrays: biotechnology's discovery platform for functional genomics. *Trends in Biotechnology*.

[9] Blencowe B. J., Ahmad S., Lee L. J. (2009). Current-generation high-throughput sequencing: deepening insights into mammalian transcriptomes. *Genes & Development*.

[10] Geng Y. T., Jiang J. T., Wu C. P. (2018). Function and clinical significance of circRNAs in solid tumors. *J. Hematol. Oncol*.

[11] Han B., Chao J., Yao H. H. (2018). Circular RNA and its mechanisms in disease: from the bench to the clinic. *Pharmacology & therapeutics Part C, Clinical pharmacology and therapeutics*.

[12] Zhang Z. K., Xie Q., He D. M. (2018). Circular RNA: new star, new hope in cancer. *Bmc Cancer.*.

[13] Li Y. W., Zheng F. X., Xiao X. Y. (2017). CircHIPK3 sponges miR-558 to suppress heparanase expression in bladder cancer cells. *EMBO Reports*.

[14] Bai N., Peng E., Qiu X. (2018). circFBLIM1 act as a ceRNA to promote hepatocellular cancer progression by sponging miR-346. *J. Exp. Clin. Cancer Res*.

[15] Liang H. F., Zhang X. Z., Liu B. G., Jia G. T., Li W. L. (2017). Circular RNA circ-ABCB10 promotes breast cancer proliferation and progression through sponging miR-1271. *American Journal of Cancer Research*.

[16] Liu Y. X., Dong Y. Y., Zhao L. P., Su L. H., Luo J. (2018). Circular RNA-MTO1 suppresses breast cancer cell viability and reverses monastrol resistance through regulating the TRAF4/Eg5 axis. *International Journal of Oncology*.

[17] Yang Z., Wu L., Wang A. (2017). dbDEMC 2.0: updated database of differentially expressed miRNAs in human cancers. *Nucleic Acids Res*.

[18] Fan C., Lei X., Fang Z., Jiang Q., Wu F. X. (2018). CircR2Disease: a manually curated database for experimentally supported circular RNAs associated with various diseases. *Database (Oxford)*.

[19] Zhao Z., Wang K., Wu F. (2018). circRNA disease: a manually curated database of experimentally supported circRNA-disease associations. *Cell Death Dis*.

[20] Salmena L., Poliseno L., Tay Y., Kats L., Pandolfi P. P. (2011). A _ceRNA_ Hypothesis: The Rosetta Stone of a Hidden RNA Language?. *Cell*.

[21] Gong Y., Mao J., Wu D. I. (2018). Circ-ZEB1.33 promotes the proliferation of human HCC by sponging miR-200a-3p and upregulating CDK6. *Cancer Cell Int.*.

[22] Deng G., Mou T., He J. (2020). Circular RNA circRHOBTB3 acts as a sponge for miR-654-3p inhibiting gastric cancer growth. *J. Exp. Clin. Cancer Res.*.

[23] He R., Liu P., Xie X. (2017). circGFRA1 and GFRA1 act as ceRNAs in triple negative breast cancer by regulating miR-34a. *J. Exp. Clin. Cancer Res*.

[24] Kanehisa M., Goto S. (2000). KEGG: Kyoto encyclopedia of genes and genomes. *Nucleic Acids Research*.

[25] Swain S. M., Miles D., Kim S. B. (2020). Pertuzumab, trastuzumab, and docetaxel for HER2-positive metastatic breast cancer (CLEOPATRA): end-of-study results from a double-blind, randomised, placebo-controlled, phase 3 study. *Lancet Oncology.*.

[26] Oh D. Y., Bang Y. J. (2020). HER2-targeted therapies -- a role beyond breast cancer. *Nature Reviews Clinical Oncology.*.

[27] Murthy R. K., Loi S., Okines A. (2020). Tucatinib, Trastuzumab, and Capecitabine for HER2-positive metastatic breast cancer. *New England Journal of Medicine.*.

[28] Modi S., Saura C., Yamashita T. (2020). Trastuzumab Deruxtecan in previously treated HER2-positive breast cancer. *New England Journal of Medicine.*.

[29] Wu J. C., Liu S., Xiang Y., Qu X. Z., Xie Y. J., Zhang X. W. (2019). Bioinformatic Analysis of Circular RNA-Associated ceRNA Network Associated with Hepatocellular Carcinoma. *Biomed Res. Int*.

[30] Liu F., Zhang H., Xie F. (2020). Hsa_circ_0001361 promotes bladder cancer invasion and metastasis through miR-491-5p/MMP9 axis. *Oncogene*.

[31] Song W., Fu T. (2019). Circular RNA-associated competing endogenous RNA network and prognostic Nomogram for patients with colorectal cancer. *Front. Oncol.*.

[32] Aiello N. M., Brabletz T., Kang Y., Angela Nieto M., Weinberg R. A., Stanger B. Z. (2017). Upholding a role for EMT in pancreatic cancer metastasis. *Nature*.

[33] Angela Nieto M., Huang R. Y.-J., Jackson R. A., Thiery J. P. (2016). EMT: 2016. *Cell*.

[34] Kahlert U. D., Joseph J. V., Kruyt F. A. E. (2017). EMT- and MET-related processes in nonepithelial tumors: importance for disease progression, prognosis, and therapeutic opportunities. *Molecular Oncology.*.

[35] Angela N. M. (2017). Context-specific roles of EMT programmes in cancer cell dissemination. *Nature Cell Biology.*.

[36] Caramel J., Papadogeorgakis E., Hill L. (2013). A Switch in the Expression of Embryonic EMT-Inducers Drives the Development of Malignant Melanoma. *Cancer Cell*.

[37] Exposito-Villen A., E Aranega A., Franco D. (2018). Functional Role of Non-Coding RNAs during Epithelial-To-Mesenchymal Transition. *Non-coding RNA*.

[38] David C. J., Massague J. (2018). Contextual determinants of TGF*β* action in development, immunity and cancer. *Nat. Rev. Mol. Cell Biol.*.

[39] Derynck R., Budi E. H. (2019). Specificity, versatility, and control of TGF-*β* family signaling. *Science Signaling*.

[40] Zhang Y. E. (2017). Non-Smad signaling pathways of the TGF-*β* family. *Perspectives in biology*.

[41] Hoxhaj G., Manning B. D. (2020). The PI3K-AKT network at the interface of oncogenic signalling and cancer metabolism. *Nature Reviews. Cancer*.

[42] Schmid P., Abraham J., Chan S. (2020). Capivasertib plus paclitaxel versus placebo plus paclitaxel as first-line therapy for metastatic triple-negative breast cancer: the PAKT trial. *Journal of Clinical Oncology*.

[43] Guo B., Wu S., Zhu X. (2020). Micropeptide CIP2A-BP encoded by LINC00665 inhibits triple-negative breast cancer progression. *Embo Journal*.

[44] Castagnoli L., Cancila V., Cordoba-Romero S. L. (2019). WNT signaling modulates PD-L1 expression in the stem cell compartment of triple-negative breast cancer. *Oncogene*.

[45] Fu M., Chen C.-W., Yang L.-Q. (2020). MicroRNA-103a-3p promotes metastasis by targeting TPD52 in salivary adenoid cystic carcinoma. *International Journal of Oncology*.

[46] Shi R., Wu P., Liu M., Chen B., Cong L. (2020). Knockdown of lncRNA *PCAT6* Enhances Radiosensitivity in Triple-Negative Breast Cancer Cells by Regulating *miR-185-5p/ TPD52* Axis. *Oncotargets and Therapy*.

[47] Shuai Y., Ma Z., Liu W. (2020). TEAD4 modulated LncRNA MNX1-AS1 contributes to gastric cancer progression partly through suppressing BTG2 and activating BCL2. *Mol. Cancer*.

[48] Chen Z., Chen X., Lu B. (2020). Up-regulated LINC01234 promotes non-small-cell lung cancer cell metastasis by activating VAV3 and repressing BTG2 expression. *J. Hematol. Oncol.*.

[49] Yu G., Zhou H., Yao W., Meng L., Lang B. (2019). lncRNA TUG1 Promotes Cisplatin Resistance by Regulating CCND2 via Epigenetically Silencing miR-194-5p in Bladder Cancer. *Molecular Therapy-Nucleic Acids*.

[50] Shao J., Zhu W., Ding Y. (2019). Phosphorylation of LIFR promotes prostate cancer progression by activating the AKT pathway. *Cancer Letters*.

[51] Woosley A. N., Dalton A. C., Hussey G. S. (2019). TGF*β* promotes breast cancer stem cell self-renewal through an ILEI/LIFR signaling axis. *Oncogene*.

[52] Park G. B., Kim D. (2019). MicroRNA-503-5p inhibits the CD97-mediated JAK2/STAT3 pathway in metastatic or paclitaxel-resistant ovarian cancer cells. *Neoplasia*.

[53] Xu K., Chen G., Qiu Y. (2017). miR-503-5p confers drug resistance by targeting PUMA in colorectal carcinoma. *Oncotarget*.

[54] Sun Q., Li Q., Xie F. (2019). LncRNA-MALAT1 regulates proliferation and apoptosis of ovarian cancer cells by targeting miR-503-5p. *Oncotargets and Therapy*.

[55] Li J.-P., Xiang Y., Fan L.-J., Yao A., Li H., Liao X.-H. (2019). Long noncoding RNA H19 competitively binds miR-93-5p to regulate STAT3 expression in breast cancer. *Journal of Cellular Biochemistry*.

[56] Huang W., Yang Y., Wu J. (2020). Circular RNA cESRP1 sensitises small cell lung cancer cells to chemotherapy by sponging miR-93-5p to inhibit TGF-*β* signalling. *Cell Death and Differentiation.*.

[57] Wang C., Li Q., He Y. (2020). MicroRNA-21-5p promotes epithelial to mesenchymal transition by targeting SRY-box 17 in endometrial cancer. *Oncology Reports.*.

